# Zika Outbreak Emergency Preparedness and Response of Malaysian Private Healthcare Professionals: Are They Ready?

**DOI:** 10.3390/microorganisms7030087

**Published:** 2019-03-19

**Authors:** Kingston Rajiah, Mari Kannan Maharajan, Pua Yin Yin, Yap Wei Yee, Wong Wan Lin, Chew Hui Kean

**Affiliations:** 1 Department of Pharmacy Practice, School of Pharmacy, International Medical University, Kuala Lumpur 57000, Malaysia; MariKannan@imu.edu.my; 2 Department of Pharmacy, School of Pharmacy, International Medical University, Kuala Lumpur 57000, Malaysia; yinyin1311@gmail.com (P.Y.Y.); vinny.yap@gmail.com (Y.W.Y.); ww11094@gmail.com (W.W.L.); huikeanchew@gmail.com (C.H.K.)

**Keywords:** Zika, Malaysia, health care providers, epidemic

## Abstract

Zika virus has been declared as a public health emergency of international concern. The Center for Disease Control and Prevention has issued guidelines reminding healthcare workers about the importance of taking steps to prevent the spread of Zika virus, how to test and isolate patients suspected of carrying the Zika virus, and how to protect themselves from infection. Therefore, it is of utmost importance for healthcare professionals to be fully aware of Zika virus preparedness, and response measures should an outbreak occur in Malaysia in order to quickly and efficiently contain the outbreak, ensure the safety of individual or healthcare personnel safety, as well as to prevent further spreading of the disease. This research aims to show how prepared Malaysian healthcare professionals are against Zika virus and how well can they respond during an outbreak. In total, 504 healthcare professionals (128 general practitioners, 215 community pharmacists, 161 nurses) from private health clinics were the target population of the four states of Malaysia where Zika cases suspected. The sample size of each category was calculated by using a formula for estimating the population proportion. An additional 10% of the calculated sample size was added to compensate the non-response rate. The Center For Disease Control and Prevention and World Health Organisation provided a checklist to assess how prepared healthcare professionals are for an Zika outbreak. This checklist was modified to a questionnaire in order to assess health care professionals’ preparedness and response to the Zika outbreak. Community pharmacists are still lacking in their preparedness and perceived response to the Zika outbreak compared to the general practitioners in the private sector. Hence community pharmacists should attend training given by the Ministry of Health Malaysia as a continuing education, which may help them to respond during a Zika outbreak.

## 1. Introduction

Epidemics of infectious diseases are a major concern for global health [1] and the recent epidemic of Zika virus Brazilhas gained attention globally [2,3,4,5], due to its complications associated with congenital malformations [6]. The recent outbreaks suggest that Zika virus, one of the arboviruses transmitted by *Aedes aegypti* mosquitoes, is mainly spread all over tropical and sub-tropical counties worldwide [7]. Presently, there is no treatment or preventive vaccine to manage the consequences of Zika virus infection [8].

Organizations such as the Centers for Disease Control and Prevention (CDC) in United States, World Health Organisation (WHO), United Nations, including the United Nations Educational, Scientific and Cultural Organization (UNESCO) and the United Nations International Children’s Emergency Fund (UNICEF) have come up with numerous initiatives for preparedness and response purposes, and for the prevention and control of the Zika virus epidemics [4,5,9,10,11]. The impact of the Zika outbreak may affect healthcare professionals. Therefore, their preparedness and response measures are of paramount importance to prevent further outbreaks, especially in the tropical regions, where *Aedes* spp mosquitoes mainly exist. With Zika virus infection manifesting as an evolving pandemic concern, it is crucial to focus on the preparedness and readiness of healthcare professionals in managing a Zika outbreak.

Healthcare professionals, are under an enormous risk of getting exposed to the infections, more than any other individuals, as they are in contact with the infected individuals. Especially, during any outbreak, they may need to work with a healthcare team [12] as their exposure to the Zika virus infection during patient care may put them under the highest risk of contracting the infection. Their awareness and preparedness in managing the risk of the Zika outbreak is very important to prevent further spread of this infectious disease.

The Asian region is susceptible to epidemic Zika transmission because of various reasons like; widespread distribution of relevant mosquito vectors, the large amount of travel to, and from, Zika-affected areas, local conditions conducive to transmission, and limited health resources [13,14]. Zika virus infections have affected Southeast Asian countries, such as Indonesia [15], Singapore [16], Vietnam [17], and Thailand [18], and Microcephaly, associated with Zika infection [19], has been also reported in Southeast Asian countries. Previous literature has furthered the knowledge, attitude, practice, and vigilance of healthcare professionals from Malaysia and some Southeast Asian countries [20,21,22,23,24,25]. However preparedness and response studies among healthcare professionals is still lacking in Malaysia and other Southeast Asian countries.

Malaysia is located in Southeast Asia, where the Aedes mosquitoes thrive and spread disease like dengue [26]. Suspected Zika cases have been reported in four states (Johor, Penang, Sarawak, and Selangor) in Malaysia and hence it is important to focus on the preparedness of healthcare professionals of Malaysia. With help from WHO Western Pacific Regional Office, Malaysia has developed its own Zika virus infection task forces, emergency operation centres, and regional responses [11], including Zika virus simulation exercises [2,3,4,5]. With the propensity of such activities, healthcare professionals have been provided information on Zika. Therefore, the aim of this study was to assess health care professionals’ preparedness and response towards Zika outbreak to understand how Malaysian healthcare professionals are prepared against the infection and how well can they respond in case of an outbreak.

## 2. Materials and Methods

### 2.1. Subjects and Survey Design

This study was conducted among private healthcare professionals in Malaysia between June 2017 and December 2017. Among the private Malaysian healthcare professionals, three categories (general practitioners in clinical, community pharmacists, and nurses in clinics) were considered in the study as these are the professionals in the Zika virus front-line. The samples were from four states (Johor, Penang, Sarawak, Selangor) in Malaysia, where the suspected cases of Zika virus infection were reported [27]. In total, 504 private healthcare professionals (128 general practitioners, 215 community pharmacists, 161 nurses) were after the sample size calculated from those four states. The sample size of each category was calculated by using a formula for estimation of unknown population proportion [28]. Ten percent of the calculated sample size was added to compensate for the non-response rate [29]. Therefore, the minimum sample size required for each category was 78 general practitioners, 172 community pharmacists, and 145 nurses. A convenient sampling method was used to select the sample. Participants who were willing to give their informed consent to participate and those who have been in practice for more than 6 months during the study period were included. Foreign health care professionals, public, healthcare professionals of Malaysia, and those who were unable to communicate were excluded. A total of 445 healthcare professionals (100 general practitioners, 193 community pharmacists, 152 nurses) from four states was approached in this study.

### 2.2. Questionnaire Design

There is a checklist provided by the CDC and Department of Health and Environmental Control (DHEC) [30,31] to assess how prepared healthcare professionals are for the Zika outbreak. This checklist was modified to a questionnaire in order to assess health care professionals’ preparedness and perceived response if there was a Zika outbreak. Prior to the study, this questionnaire was piloted on 20 private healthcare professionals from four states (five from each state mentioned above) in Malaysia. No modifications were required following the evaluation. The Cronbach’s alpha was 0.78 for preparedness and 0.74 for perceived response. The overall Cronbach’s alpha was 0.76; indicating that all of the items included make a valid contribution. The questionnaire was a ‘Yes’ or ‘No’ type comprised of 30 questions. The statistical computer package, IBM SPSS, Version 22 was used to perform quantitative analysis on the collected data. In order to perform analysis of data, it was necessary to code the response variables, i.e., yes = 1, no = 0. Data cleaning was carried out throughout the data entry process. The analysis of the data was performed through descriptive statistics, such as frequencies for each of the variables. One-way analysis of variance (ANOVA) was used to determine the significant differences of each variable between the groups, followed by Tukey’s post hoc test to determine the differences of each variable within the groups. The threshold for statistical significance was *p* ≤ 0.05.

### 2.3. Ethics

The study was approved by the International Medical University Research and Ethics Joint Committee, project number BP I-01-14 (23)2017. The names and identity card numbers of participants were excluded to ensure anonymity and confidentiality. Before the survey, a study information sheet was shown, and individual verbal or written consent was obtained.

## 3. Results

A total of 422 healthcare professionals completed the survey, resulting in a response rate 94.8 percent. More than 50% of the respondents were female. The majority of the respondents were in the age range between 31 and 40 years. In terms of experience, 50.23% of the respondents had at least 10 years during the study period. As a multi-ethnic country, the respondents’ ethnicity was comprised of 33% Malay, 39% Chinese, and 26% Indians. Table 1 shows the statistical summary of the demographic details of the respondents.

The mean scores presented in Table 2 and Table 3 were obtained by calculating the total response for each item in the questionnaire and the total sample participated in each category. Table 2 presented the health care professionals’ preparedness for a Zika outbreak. The data revealed that most of the respondents were prepared for the Zika outbreak. Yet, there were significant differences between healthcare professionals on some of the items asked in the questionnaire. The nurses found it difficult to access the research literature on Zika virus management, with a low mean score for this statement (0.31 ± 0.34), while general practitioners found it easy to access the literature with the highest mean score among the other groups (0.69 ± 0.31), with the significant value *p* = 0.03. Community Pharmacists felt that they have not participated in educational activities related to Zika virus preparedness on a consistent basis (continuing professional development, education classes, seminars, or conferences) with a low mean score (0.36 ± 0.31), whereas the general practitioners (0.61 ± 0.35) had the highest mean scores and felt they have participated in such activities on a regular basis *p* = 0.002. Community Pharmacists considered themselves, not prepared for the management of Zika virus, with a low mean score (0.35 ± 0.27), whereas the general practitioners in this study considered themselves prepared for the management of Zika virus with the highest mean score (0.72 ± 0.27) *p* = 0.002.

Table 3 presents the health care professionals’ perceived responses to the Zika outbreak. The data revealed that most of the respondents perceived postive responses towards theie preparedness for the Zika outbreak. Yet, there were significant differences found between health care professionals’ perceived response towards Zika outbreak on some of the items asked in the questionnaire. Community pharmacists were not confident in identifying the signs and symptoms of Zika virus (0.46 ± 0.44) and providing patient education on the Zika virus (0.71 ± 0.48), with low mean scores for these statements *p* = 0.05. However, the general practitioners had highest mean scores for these statements.

Sub-group one-way analysis of variance tests (Table 4) did not reveal any significant differences in mean scores for preparedness among the health care professionals regarding age, gender, profession, and ethnicity. However, there was a significant difference in mean scores for preparedness by level of experience among healthcare professionals. Tukey’s post hoc test revealed that among the experience level of >5 to 10 years, the general practitioners mean score 14.52 ± 3.12 was significantly higher than those of the other healthcare professionals; nurses 14.21 ± 3.63 (*p* = 0.04) and pharmacists 13.87 ± 3.42 (*p* = 0.03).

Sub-group one-way analysis of variance test (Table 5) revealed significant differences in perceived response scores among healthcare professionals by age. Tukey’s post hoc test revealed that, among the age group of 20–30 years, the nurses mean score (14.02 ± 4.95) was approximately 25% higher than those of pharmacists (11.22 ± 4.34), which was significant *p* = 0.018. Tukey’s post hoc test also revealed that there was a significant difference in mean scores of the perceived response by level of experience among the healthcare professionals. The experience level up to 5 years, the nurses mean score (14.53 ± 4.09) was approximately 25% higher than those of pharmacists (12.78 ± 4.71), which was significant *p* = 0.003. No significant differences in any other demographic factors were observed.

The total mean scores of healthcare professionals’ preparedness and perceived response to the Zika outbreak is given in Table 6. The overall preparedness for a Zika outbreak, as measured by the total mean score, of general practitioners (13.21 ± 4.14) was approximately 15% higher than those of the other healthcare professionals. One-way ANOVA test revealed significant differences between general practitioners and pharmacists, *p* = 0.001. The overall perceived response to the Zika outbreak, as measured by the total mean score, of the general practitioners (9.28 ± 2.46) was approximately 25% higher than those of the other healthcare professionals. One-way ANOVA revealed significant differences between general practitioners and nurses, *p* = 0.01.

## 4. Discussion

Widespread efforts and worldwide funds have been invested in the last two years to reinforce the preparedness and response of healthcare professionals in order to manage the Zika virus infection. Various types of interventions have been implemented by international and national authorities in order to facilitate patients with suspected and actual Zika virus infection. Although various measures are ongoing in Malaysia to prevent the Zika virus, recent studies [32,33] revealed that it is essential to assess the preparedness and response of healthcare professionals in this country. 

As the female ratio is more than half in Malaysia, the female population of respondents was higher in this study as well. Most of the healthcare professionals were low-middle-aged and have 10 years or less experience in their field. This shows that, the private health care professionals in Malaysia are relatively young compared to the healthcare professionals in public sectors [34]. The ethnic distribution of healthcare professionals is equal among Malay, Chinese, and Indian, which are the three major ethnic groups in Malaysia. 

Though healthcare professionals are prepared for a Zika virus infection outbreak, nurses have difficulty in accessing the research literature on Zika virus infection, which shows that either the nurses were too busy with patients or they must have limited access to literatures. However, the Ministry of Health Malaysia advise that nurses should have regular participation in a journal club [35]. Community pharmacists’ lack of participation in continuing education programs reduced their score in terms of Zika virus infection preparedness. Community pharmacists should be strongly encouraged to attend compulsory continuing education similar to nurses in Malaysia. The reason community pharmacists consider themselves not prepared for the management of Zika virus infection, may be due to the same reasons discussed, that they are busy dispensing medications on the counter. Attending mandatory continuing education may resolve this problem. Government agencies have spent a significant amount and resources to educate medical staff and to make information about the Zika virus infection epidemic publicly available [36]. However, the resources have mainly targeted primary care staff in government services. The down flow of information about the Zika virus infection might have not been disseminated to all the private departments. Our findings, therefore, advocate that existing systems of transmission may not be an effective way to reach private sectors, which would otherwise not have the access to the information.

Healthcare professionals perceived a positive response towards the Zika outbreak. However, community pharmacists were not confident in identifying the signs and symptoms of Zika virus and providing patient education for the same. The reason for not being confident expressed by community pharmacists may be due to a lack of information on Zika virus infection preparedness. The general practitioners’ and nurses’ perceived responses in identifying the signs and symptoms of Zika virus infection and providing patient education were higher, which may be due to the training programs they attend. 

Working experience has had a huge impact on the preparedness of healthcare professionals. General practitioners, with the experience of more than five years were more prepared than other healthcare professionals with the same experience level. This may be because general practitioners have undergone training conducted by the Ministry of Health Malaysia, through a simulated exercise to avert the possibility of the spread of Zika virus. This kind of simulated training should be given to all healthcare professionals, especially community pharmacists in Malaysia. 

Age played a key role in health care professionals’ perceived response towards the Zika virus. Nurses, who were between 20–30 years old, perceived they can respond better than community pharmacists with the same age range. This indicates that, although nurses are busy with their daily activities, they participate in continual education whenever there is an emergency outbreak in the country, whereas, the community pharmacists do not. Experience also played an important role in the health care professionals’ perceived responses towards the Zika virus. General practitioners who had less than five years experience perceived that they can respond better than community pharmacists with the same years of experience. This may be, because as recent graduates, the training and simulation exercise were fresh in the minds of general practitioners. But the community pharmacists again proved that, the lack of training and simulation exercise regarding Zika virus infection had caused their response towards Zika virus not in parity with other healthcare professionals. 

The total mean score for preparedness and responses to Zika virus infection was positive among the health care professionals, although there was a range of variance in scores. Probable reasons may be the fact that the preparedness and response programme have been already introduced and executed by the national emergency authority of the Ministry of Health, Malaysia. However, community pharmacists in the private sector still lack in preparedness and perceived response to Zika virus infection. According to the CDC, community pharmacists also have to be prepared for Zika virus. Hence, community pharmacists should attend training given by the Malaysia Epidemic Intelligence Program (EIP) as a continuing education program.

### Limitation

Healthcare professionals in the public sector were not included in this study as access to public hospitals could was not approved within the stipulated timeframe during the study. Future studies should focus on public healthcare professionals. Convenience sampling may cause selection bias and effects outside the control of the researchers. Therefore, caution should be exercised in generalizing these findings. All data were self-reported, and no cross verification of actual competence of methods and techniques were made.

## 5. Conclusions

Healthcare professionals in the Malaysian private sector perceived that they are ready and can respond at any moment in case of Zika outbreak. However, community pharmacists in the private sector are still lacking in their preparedness and perceived that they cannot respond efficiently in case there is a Zika outbreak. As the Ministry of Health, Malaysia, is vigilant and continually conducting preventive measures programs. Participation of community pharmacists may improve their preparedness and response towards any emergency outbreak of Zika virus infection in Malaysia.

## Figures and Tables

**Table 1:** Healthcare professionals’ demographic information (*n* = 422).

Demographic Characteristic		*n*	%
**Age in years**	20–30	96	22.74
	31–40	188	44.54
	41–60	138	32.54
**Gender**	Male	203	48.10
	Female	219	51.89
**Profession**	General practitioner	82	19.43
	Community pharmacist	188	44.54
	Nurse	152	36.01
**Ethnicity**	Malay	143	33.88
	Chinese	167	39.57
	Indian	112	26.54
**Experience in years**	Up to 5 years	72	17.06
	6–10 years	212	50.23
	More than 10 years	138	32.70

**Table 2:** Healthcare professionals’ preparedness for Zika outbreak.

Preparedness	Mean Scores for Each Items (±SD)			
	General Practitioners	Nurses	Pharmacists	*p* Value
I know all the information about Zika virus preparedness related to my community needs.	0.68 (±0.38)	0.70 (±0.32)	0.71 (±0.35)	0.143
I am aware of the obstacles in Zika virus preparedness related to my community.	0.71 (±0.39)	0.78 (±0.31)	0.72 (±0.34)	0.289
I am aware of educational classes on Zika virus preparedness that relate specifically to my community situation.	0.70 (±0.31)	0.60 (±0.38)	0.55 (±0.30)	0.312
I am aware of the programs about Zika virus preparedness and management that are offered by Ministry of Health Malaysia.	0.65 (±0.34)	0.68 (±0.32)	0.50 (±0.32	0.587
I read journal articles related to Zika virus preparedness.	0.81 (±0.28)	0.64 (±0.35)	0.68 (±0.38)	0.243
In case of an emergency situation, there is sufficient support from local officials in this region.	0.52 (±0.31)	0.60 (±0.39)	0.58 (±0.32)	0.415
I know who to contact (chain of command) in disaster situations in my community.	0.68 (±0.34)	0.71 (±0.30)	0.41 (±0.36)	0.816
I find that, the research literature on Zika virus management is easily accessible.	0.69 (±0.31)	0.31 (±0.34)	0.53 (±0.36)	0.032 *
I participate in educational activities dealing with Zika virus VIRUS preparedness on a regular basis (continuing education classes, seminars, or conferences).	0.61 (±0.35)	0.58 (±0.33)	0.36 (±0.31)	0.002 *
I have participated in emergency plan drafting and emergency planning for Zika virus situations in my community.	0.33 (±0.28)	0.38 (±0.32)	0.31 (±0.34)	0.481
Upon admission, a relevant exposure history should be taken including exposure criteria of whether the patient has resided in or travelled to a country.	0.93 (±0.10)	0.82 (±0.23)	0.79 (±0.29)	0.564
In case of bioterrorism/biological attacks, I know how to use personal protective equipment.	0.78 (±0.37)	0.62 (±0.34)	0.78 (±0.32)	0.518
In case of bioterrorism/biological attacks I know how to execute decontamination procedures.	0.79 (±0.36)	0.46 (±0.31)	0.48 (±0.37)	0.283
I am familiar with accepted triage principles used in emergency situations.	0.78 (±0.24)	0.52 (±0.38)	0.49 (±0.31)	0.490
In a case of emergency I know how to perform isolation procedures to minimize the risks of community exposure.	0.89 (±0.28)	0.52 (±0.31)	0.48 (±0.39)	0.383
I am familiar with the local emergency response system for Zika virus.	0.79 (±0.34)	0.45 (±0.37)	0.51 (±0.32)	0.167
I would be considered a key leadership character in my community in Zika outbreak.	0.52 (±0.38)	0.39 (±0.32)	0.31 (±0.38)	0.441
I consider myself prepared for the management of Zika virus.	0.72 (±0.27)	0.54 (±0.28)	0.35 (±0.27)	0.002 *

* *p* < 0.05.

**Table 3:** Healthcare professionals’ perceived response for Zika outbreak.

Perceived Response	Mean Scores for Each Items (±SD)			
	General Practitioners	Nurses	Pharmacists	*p* Value
I am confident in providing patient education on Zika virus.	0.79 (±0.42)	0.68 (±0.51)	0.48 (±0.48)	0.049 *
I am able to identify the signs and symptoms of Zika virus.	0.89 (±0.32)	0.75 (±0.50)	0.46 (±0.44)	0.049 *
I am familiar with the scope of my role in Zika virus as a healthcare provider.	1.00 (±0.00)	0.81 (±0.51)	0.45 (±0.40)	0.541
I am reasonably confident in my abilities in Zika virus as a member of a healthcare team.	0.84 (±0.37)	0.50 (±0.51)	0.69 (±0.47)	0.365
I would feel confident in my abilities as a direct care provider or first responder in Zika virus.	0.79 (±0.42)	0.59 (±0.50)	0.42 (±0.50)	0.407
I can care for Zika virus patients independently without any supervision.	0.47 (±0.51)	0.27 (±0.46)	0.25 (±0.44)	0.743
I can manage the common symptoms and reactions of Zika virus.	0.90 (±0.32)	0.71 (±0.50)	0.41 (±0.46)	0.488
I would feel confident implementing emergency plans, evacuation procedures, and similar functions.	0.53 (±0.51)	0.45 (±0.51)	0.27 (±0.45)	0.329
I can identify possible indicators of mass exposure evidenced by a clustering of patients with similar symptoms.	0.74 (±0.45)	0.50 (±0.51)	0.48 (±0.50)	0.642
As a healthcare provider, I would feel confident as a manager or coordinator of a shelter.	0.58 (±0.51)	0.45 (±0.51)	0.38 (±0.49)	0.565
I am ready to participate in peer evaluation of skills on Zika virus.	0.40 (±0.42)	0.35 (±0.51)	0.50 (±0.50)	0.438
I am familiar with how to perform focused health assessment for Zika virus.	0.68 (±0.48)	0.36 (±0.49)	0.29 (±0.46)	0.231

* *p* < 0.05.

**Table 4:** Differences in preparedness among healthcare professionals by demographic profile.

Age in Years	General Practitioners (±SD)	Nurses (±SD)	Pharmacists (±SD)	*p* Value
20–30	12.78 ± 3.21	13.43 ± 2.42	13.21 ± 3.54	0.786
31–40	12.67 ± 3.32	13.54 ± 2.67	13.36 ± 3.52	0.723
41–60	11.64 ± 3.34	12.29 ± 3.69	11.89 ± 3.34	0.612
**Gender**				
Male	13.67 ± 3.52	13.57 ± 3.82	13.21 ± 3.62	0.697
Female	13.82 ± 3.53	13.21 ± 3.71	13.02 ± 3.58	0.634
**Ethnicity**				
Malay	13.72 ± 3.41	13.42 ± 3.15	13.97 ± 3.28	0.616
Chinese	13.21 ± 3.18	13.84 ± 3.42	12.98 ± 3.71	0.673
Indian	13.62 ± 3.64	13.21 ± 3.51	12.87 ± 3.47	0.707
**Experience in Years**				
Up to 5 years	13.51 ± 3.71	13.58 ± 3.17	13.21 ± 3.74	0.732
6–10 years	14.52 ± 3.12	14.21 ± 3.63	13.87 ± 3.42	<0.049 ^a,b^
More than 10 years	13.22 ± 3.45	13.45 ± 3.62	13.13 ± 3.62	0.715

Data expressed are total mean scores ± standard deviation (SD). One-way ANOVA was used. ^a^ = *p* < 0.05 for the difference between general practitioners and pharmacists (one-way ANOVA with the Tukey’s post hoc test).^b^ = *p* < 0.05 for the difference between general practitioners and nurses (one-way ANOVA with the Tukey’s post hoc test).

**Table 5:** Differences in perceived response among healthcare professionals by demographic profile.

Demographic Characteristic	General Practitioners (±SD)	Nurses (±SD)	Pharmacists (±SD)	*p* Value
**Age in Years**				
20–30	13.81 ± 4.11	14.02 ± 4.95	12.87 ± 3.28	0.018 ^a^
31–40	14.36 ± 4.76	13.94 ± 4.22	12.45 ± 3.54	0.703
41–60	14.45 ± 4.62	14.01 ± 4.47	12.79 ± 3.06	0.825
**Gender**				
Male	13.91 ± 3.98	14.04 ± 4.02	13.34 ± 3.87	0.692
Female	13.80 ± 4.28	14.05 ± 3.76	12.79 ± 4.07	0.707
**Ethnicity**				
Malay	14.34 ± 4.26	14.68 ± 4.06	13.87 ± 4.36	0.434
Chinese	13.87 ± 3.84	14.73 ± 4.10	13.49 ± 4.15	0.569
Indian	13.97 ± 4.07	14.78 ± 4.12	13.58 ± 4.24	0.385
**Experience in Years**				
Up to 5 years	13.47 ± 3.81	14.53 ± 4.09	12.78 ± 4.71	0.003^a^
6–10 years	14.58 ± 3.92	13.73 ± 4.20	12.54 ± 4.90	0.426
More than 10 years	14.35 ± 4.38	13.68 ± 4.31	12.68 ± 4.75	0.351

Data expressed are total mean scores ± standard deviation (SD). One-way ANOVA was used. ^a^ = *p* < 0.05 for the difference between pharmacists and nurses (one-way ANOVA with the Tukey’s post hoc test).

**Table 6:** Difference in preparedness score and perceived response score among healthcare professionals.

Healthcare Professionals	General Practitioners (±SD)	Nurses (±SD)	Pharmacists (±SD)	*p* Value
**Preparedness**	13.21 ± 4.14	9.76 ± 4.32	8.92 ± 4.98	0.001 ^a^
**Perceived response**	9.28 ± 2.46	5.64 ± 2.73	6.53 ± 2.29	0.012 ^b^

Data are expressed as mean ± standard deviation (SD). One-way ANOVA was used. ^a^ = *p* < 0.05 for the difference between general practitioners and pharmacists (one-way ANOVA with the Tukey’s post hoc test). ^b^ = *p* < 0.05 for the difference between nurses and pharmacists (one-way ANOVA with the Tukey’s post hoc test).
